# The Unknown Unknowns: Recovering Gamma-Delta T Cells for Control of Human Immunodeficiency Virus (HIV)

**DOI:** 10.3390/v12121455

**Published:** 2020-12-17

**Authors:** Shivkumar Biradar, Michael T. Lotze, Robbie B. Mailliard

**Affiliations:** 1 Department of Infectious Diseases and Microbiology, University of Pittsburgh, Pittsburgh, PA 15261, USA; SSB38@pitt.edu; 2 Departments of Surgery, Immunology, and Bioengineering, University of Pittsburgh, Pittsburgh, PA 15261, USA; lotzemt@upmc.edu

**Keywords:** gamma delta T cell, Vδ2, Vδ1, HIV, immunotherapy

## Abstract

Recent advances in γδ T cell biology have focused on the unique attributes of these cells and their role in regulating innate and adaptive immunity, promoting tissue homeostasis, and providing resistance to various disorders. Numerous bacterial and viral pathogens, including human immunodeficiency virus-1 (HIV), greatly alter the composition of γδ T cells in vivo. Despite the effectiveness of antiretroviral therapy (ART) in controlling HIV and restoring health in those affected, γδ T cells are dramatically impacted during HIV infection and fail to reconstitute to normal levels in HIV-infected individuals during ART for reasons that are not clearly understood. Importantly, their role in controlling HIV infection, and the implications of their failure to rebound during ART are also largely unknown and understudied. Here, we review important aspects of human γδ T cell biology, the effector and immunomodulatory properties of these cells, their prevalence and function in HIV, and their immunotherapeutic potential.

*Reports that say that something hasn’t happened are always interesting to me, because as we know, there are known knowns; there are things we know we know. We also know there are known unknowns; that is to say we know there are some things we do not know. But there are also unknown unknowns—the ones we don’t know we don’t know. And if one looks throughout the history of our country and other free countries, it is the latter category that tends to be the difficult ones. United States Secretary of Defense Donald Rumsfeld; 12 February 2002.*

## 1. Introduction

Although conventional alpha-beta (αβ) T cells have been studied intensively as a current “known” in the field of immunology, with the basis of their recognition of processed peptides in the context of MHC molecules being well-understood, far less attention has been paid to gamma delta (γδ) T cells. This, is in part due to their relatively limited numbers in the blood and spleen, and because they reside primarily in tissues less readily accessible for study. They have important early innate immune functions, recognizing relatively conserved pathogen-associated molecules, simple metabolites, and stress ligands expressed on infected and transformed cells. They can mount rapid and direct cytolytic responses while producing cytokines and chemokines to promote the function and mobilization of other immune effector cells [1]. They are widely distributed throughout the body [2], particularly within epithelial sites, and have the capability to recognize a variety of self-, as well as non-self-antigens without regard to MHC constraint. Their prospective utilization or strategic targeting in novel immunotherapeutic approaches to treat a variety of chronic diseases is quite attractive. This is especially true in instances where classical αβ T cell immune escape may be of particular concern. However, because their frequency and function can be severely altered in the setting of diseases such as HIV, and because there are still so many “unknowns” regarding the basic biology of γδ T cells in health and disease, there are hurdles to be addressed before their therapeutic potential can be fully appreciated. In this review, we trace some of the current progress in studies related to γδ T cell biology as we explore their function, particularly in the setting of chronic HIV infection, and discuss their potential for therapeutic application.

## 2. Phenotypic and Functional Subsets of γδ T Cells

Based on TCR δ chain usage, human γδ T cells can be categorized into three major subsets: Vδ1, Vδ2, and Vδ3 T cells. Vδ1 T cells constitute less than 30% of total γδ T cells in the peripheral blood, but they are the predominant population in tissues, including the dermis, spleen, liver, gut epithelia, lung, and other mucosal sites [3]. Vδ1 T cells maintain epithelial tissue integrity, expressing classical rearranged receptors that are selected in the thymus, and function in part through recognition of the stress-inducible ligands MICA and MICB expressed by virus-infected and transformed cells, and self-glycolipids presented by CD1c/d molecules (Table 1) [4]. Vγ9Vδ2 T cells circulating in the peripheral blood and lymphatics recognize phosphoantigens produced by various microbes and transformed host cells in an MHC non-dependent fashion [1]. The third subtype of γδ T cells, the Vδ3 T cells, comprise approximately 0.6% of peripheral blood T lymphocytes, and are also found in the liver and gut (Table 1) [5,6]. Although there are no known antigens specifically identified in association with Vδ3 T cell receptor recognition, activation of this cell type has been shown to occur through their cognate interactions with MHC-class I as well as CD1d [7], an MHC-like molecule capable of presenting lipid moieties. The frequency of Vδ3 T cells is often expanded in the peripheral blood of renal and stem cell transplant recipients with cytomegalovirus reactivation [8], and in those with B cell chronic lymphocytic leukemia [9]. Similar to Vδ2 T cells, Vδ3 T cells can also act as a bridge, linking innate and adaptive branches of immunity through their modulation of B cell and dendritic cell (DC) function [10,11]. Furthermore, Vδ4, Vδ6, Vδ7, and Vδ8 T cells have been detected in the peripheral blood of lymphoma patients, serving as distinct minority subsets of γδ T cells, but their chain pairing and activation mechanisms remain unclear [12].

γδ T cells can also be categorized into different subgroups based on their functional characteristics, which include the Th1-like IFN-γ producing subset, the IL-17 producing cells, and those which function as regulatory γδ T cells. Human peripheral blood γδ T cells activated with isopentenyl pyrophosphate (IPP) can be differentiated into Th1-like cells by culturing them in the presence of recombinant (r)IL-12p70 and anti-IL-4 blocking antibodies [13]. Conversely, γδ T cells cultured in the presence of rIL-4 and anti-IL-12 blocking antibodies differentiate into Th2-like cells [13].

## 3. Ligand Recognition by γδ T Cells

**Figure 1:**
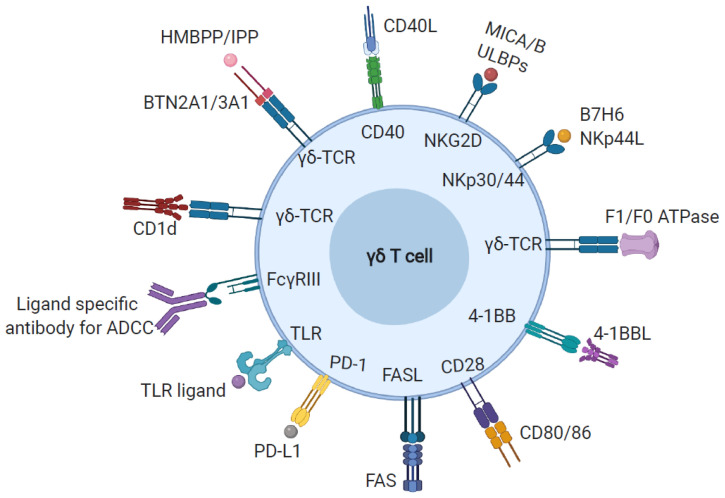
Ligand recognition by gamma delta (γδ) T cells. γδ T cells provide a wide range of immunologic functions, recognizing a diverse array of self- and non-self-ligands through their expression of various signaling receptors (depicted in the figure). Abbreviations: HMBPP: (E)-4-Hydroxy-3-methyl-but-2-enyl pyrophosphate, IPP: Isopentenyl pyrophosphate, BTN2A1: Butyrophilin 2A1, ADCC: Antibody-dependent cell-mediated cytotoxicity, TLR: Toll-like receptor, PD1: Programmed cell death protein 1, MICA/B: MHC class I chain-related protein A/B, ULBP- UL16 binding protein, NKG2D: Natural killer group 2D.

Although γδ TCRs are less diverse than αβ TCRs, they recognize a plethora of molecules such as non-peptidic metabolites of isoprenoid biosynthesis, stress molecules (MICA and MICB), heat-shock proteins, and others (Figure 1) [21]. Recognition of these molecules allows them to respond to many microbial components, as well as transformed and infected host cells, by inducing direct cytotoxicity and rapid secretion of inflammatory chemokines and cytokines [22]. The various ligands recognized by γδ T cells are explored in detail below.

### 3.1. Phosphoantigen

The breadth and variety of antigens that γδ T cells are specifically capable of recognizing have yet to be completely defined. However, their capacity to recognize non-peptidic antigens (Ag) has been studied in great depth. The predominant circulating Vγ9Vδ2 T cells, which contribute to 90–95% of the total γδ T cells in human peripheral blood, recognize phosphorylated isoprenoid precursors, collectively called phosphoantigens (pAg) [23]. The most-studied pAg in prokaryotes is (E)-4-Hydroxy-3-methyl-but-2-enyl pyrophosphate (HMBPP), an intermediate of the 2-C-methyl-d-erythritol 4-phosphate (MEP) pathway, and in eukaryotes it is IPP, an intermediate of the mevalonate pathway [23]. Besides microbial activation of γδ T cells, pAg that accumulates in transformed cells can also trigger γδ T cell activation [24]. In contrast to αβ T cells, which recognize peptide antigens presented on MHC molecules, γδ T cells recognize pAg in the context of members of the immunoglobulin superfamily members, butyrophilin 2A1 and 3A1 (BTN2A1 and BTN3A1). Thus, butyrophilin-mediated interaction activates the signaling of the Vγ9Vδ2 TCR. Specifically, BTN2A1 binds the Vγ9+ domain of the TCR, whereas BTN3A1 binds the Vδ2 and γ-chain regions on the opposite side of the TCR to mediate signaling [25]. Interestingly, pAg is thought to bind within the cytosolic portion of the butyrophilins, promoting allosteric changes [26]. This new understanding of BTN and the γδ TCR signaling pathway may facilitate the development of future γδ T cell-based immunotherapies.

### 3.2. MHC-Like Ligands

γδ T cells recognize cellular stress proteins and pathogen-associated molecules through their expression of several common natural killer (NK) cell receptors, including NKp30, NKp44, NKp46, and the C-type lectin-like receptors NKG2D, NKG2A, and NKG2C. Specifically, Vδ1 T cells recognize the MHC-like molecules CD1a, b, c, and d that present lipid Ags (glycolipids and certain microbial lipids) and are primarily expressed on professional Ag-presenting cells [27]. Tissue-resident Vδ1 T cells demonstrate reactivity to these ligands by producing several effector proteins, including IFN-γ and granulysin. γδ T cells also maintain epithelial tissue integrity by recognizing stress-induced MHC class I-related molecules MICA and MICB in an MHC-independent fashion [28].

### 3.3. Other Cell Surface and Soluble Proteins

Besides phosphoantigens, γδ T cells also recognize the mitochondrial F1/F0-ATPase-related structure expressed on the Burkitt’s lymphoma cell line, Daudi [29]. Several bacterial proteins can elicit γδ T cell responses, including staphylococcal enterotoxin A [30], toxin listeriosis O [31], tetanus toxoid from Clostridium tetani [32], and the highly virulent and immunogenic protein of Mycobacterium tuberculosis (Mtb) ESAT-6 [33]. γδ T cells can also express co-stimulatory molecules, including the tumor necrosis factor (TNF) receptor family molecules CD27, CD30, CD137 (4-1BB), and inhibitory receptors such as CD279 (PD-1), to contribute to the activation and regulation of innate and adaptive immune responses [34,35]. Moreover, Vδ2 T cells express CD16 (Fcγ receptor), which can facilitate antibody-mediated cellular cytotoxicity (ADCC) activity [36,37].

## 4. γδ T Cell Interaction with Other Immune Cells

**Figure 2:**
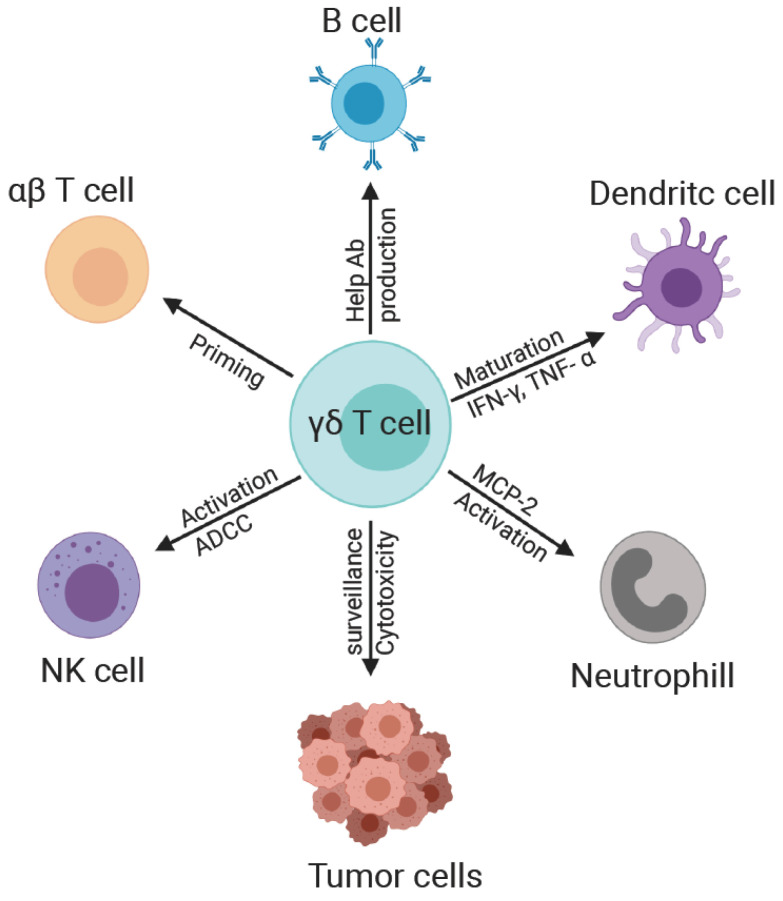
Crosstalk of γδ T cells with other immune cells. Dendritic cell: Activated γδ T cells secrete IFN-γ and TNF-α, which promote upregulation of CD86 and MHC class I molecules on DCs and increase DC secretion of IL-12p70. B cell: γδ T cells secrete IL-4, IL-10, and CXCL13, providing help to B cells for antibody production. Neutrophil: γδ T cells secrete IL-17A and CXCL8, which recruit neutrophils to inflammatory sites. NK cell: γδ T cells provide NK cells with the costimulatory signal CD137L to promote their activation and enhanced cytolytic activity. αβ T cell: Activated γδ T cells can process soluble antigens and present them in the context of both MHC class I and Class II molecules to naïve CD8+ and CD4+ αβ T cells respectively, to drive their proliferation, and differentiation. Tumor cells: γδ T cells can provide immunosurveillance and direct cytotoxicity of tumor cells.

In addition to their ability to recognize microbial and cellular stress ligands to carry out their direct effector functions, γδ T cells play an integral role in the immune system in part through their extensive crosstalk and intercellular communication activities with other immune cells (Figure 2).

### 4.1. γδ and NK Cells

Crosstalk between NK cells with γδ T cells not only impacts innate immune function, it also greatly influences the quality, nature, and outcome of subsequent adaptive immune responses [38,39,40]. Bisphosphonates, such as the FDA-approved drug zoledronate, can activate γδ T cells. NK cells interact with CD137 expressed on activated γδ T cells, resulting in the upregulation of the cytotoxic receptor NKG2D on NK cells, enhancing their capacity to recognize and kill tumors that are usually resistant to NK cytolysis [20]. In Listeria monocytogenes-infected mice, γδ T cells produce IFN-γ early during the infection, aiding in the NK cell’s capacity to mount effective innate immune responses against the intracellular pathogen [41]. It is now accepted that γδ T cells play an essential role in regulating NK cell-mediated immunity and that the absence of or dysfunction of γδ T cells can impair NK cell activation and function [42].

### 4.2. γδ and B Cells

γδ T cells play a critical role in promoting B cell maturation, antigen presentation, and antibody production, thus greatly impacting humoral immunity [43]. Activated γδ T cells can produce a variety of chemokines, including CXC-chemokine ligand 13 (CXCL13), a critical factor involved in B cell arrangement and their interaction with follicular T helper cells within lymphoid tissue, as well as germinal center formation [44]. While αβ T cells are also important for the germinal center formation and B cell production of immunoglobulins (Ig’s), mice deficient in TCRα can efficiently develop normal lymph node germinal centers and maintain the capacity to produce Ig’s, suggesting that γδ T cells play a role in providing help to support B cell function [45]. In vitro studies have demonstrated that γδ T cells in the presence of IL-4 can induce B cell activation, Ig isotype switching, and secretion of IgE [46]. Co-culturing Vδ3 T cells and B cells results in B cell maturation, characterized by upregulation of CD40, CD86, and HLA-DR surface expression on both cell types [10]. This suggests that similar to Vδ2 T cells, Vδ3 T cells can induce B cell maturation and differentiation into functional antigen presenting cells (APCs) capable of activating conventional T cells [19].

### 4.3. γδ and Monocyte/Macrophages

Monocytes and γδ T cells are rapidly recruited to the sites of infection or inflammation, and they affect each other’s ability to effectively eliminate infected cells [47,48]. Microbe-activated Vγ9Vδ2 T cells can drive the differentiation of monocytes into inflammatory DCs, promoting their antigen presentation capacity and inflammatory cytokine production [47]. Notably, the activation process is bidirectional, with increased cytotoxic activity and migration of γδ T cells occurring when they are exposed to monocytes or macrophages infected with pathogens, such as *M. tuberculosis* (Mtb) [48,49]. γδ T cells expanded in culture with the use of pAgs display potent cytotoxic activity against influenza virus-infected macrophages and promote viral clearance [50]. Moreover, in later stages of malaria infection, a γδ T cell subset producing M-CSF, CCL3, and CCL5, has been shown to be particularly important for acting on myeloid cells to prevent parasitemic recurrence [51]. 

### 4.4. γδ and Dendritic Cells

γδ T cells interact with DCs to induce their maturation in vitro [52], characterized by the upregulation of MHC molecules and co-stimulatory molecules, such as HLA-DR, CD86, and CD83 on DCs. DC maturation is contact-independent and predominantly driven by TNF-α secreted from activated γδ T cells. Phosphoantigen-mediated activation of γδ T cells induces IL-12p70 production by DC, which in turn is critical for driving the differentiation of naïve αβ T cells into IFN-γ-producing effector cells [52,53]. Besides Vδ2 T cells, Vδ1 T cells can also induce DC maturation. Tissue-resident Vδ1 T cells, interacting with CD1a, b, and c molecules expressed on immature DCs, promotes DC maturation [54]. Early during microbial infection, when there are no apparent microbe-specific CD8 T cells, these Vδ1 T cells can induce maturation of DCs and enhance their ability to present antigens to naïve CD4+ T cells. γδ T cells produce a large quantity of IFN- γ early during TB infection, which helps DCs to prime antigen specific CD8 T cells, generating protection against TB infection [55]. Recent studies suggest that human Vδ3 T cells are also capable of influencing DC maturation and cytokine production [19].

### 4.5. γδ T and αβ T Cells

In addition to producing cytokines and chemokines, human γδ T cells can impact αβ T cell function by acting as potent antigen-presenting cells [56]. While Vγ9Vδ2 T cells predominantly circulate in peripheral blood, following their activation they can express MHC class I and class II molecules, the co-stimulatory molecules CD80 and CD86, and the lymph node homing CC-chemokine receptor 7 (CCR7) [56]. Furthermore, activated human γδ T cells can process and present soluble antigens in the context of both MHC class I and Class II to naïve CD8+ and CD4+ αβ T cells respectively, to drive their activation and differentiation [57,58].

Although precise mechanisms of their antigen uptake have not been well described, Seino et al. demonstrated that activated γδ T cells can phagocytose apoptotic cells and tumor antigens, possibly utilizing the scavenger receptor CD36 in a C/EBPα (CCAAT/enhancer-binding protein α)-dependent mechanism and mount a tumor antigen-specific CD8+ T cell response [59]. Recently, Wang et al. demonstrated that exosomes isolated from allogeneic Vδ2 cells displayed impressive antitumor activity against EBV-associated tumors in humanized mice [60]. These Vδ2-derived exosomes were shown to increase the infiltration of αβ T cells and induced robust CD4+ and CD8+ T cell-mediated antitumor immunity. This fact highlights their therapeutic potential to initiate antigen-specific adaptive responses against various pathogens.

## 5. γδ T Cells in HIV-1 Infection

While γδ T cells have been described to provide protective immunity against tumors of epithelial [14,15] and hematological origin [61,62], they have also been explored in the setting of various chronic viral [16,18,63,64,65] and bacterial diseases [66,67], as well as malaria [68,69]. Furthermore, γδ T cells contribute to the pathogenesis and regulation of autoimmune diseases, including rheumatoid arthritis and psoriasis [70,71]. For the remainder of this paper, we have chosen to focus our attention on the function of these cells in the setting of chronic HIV-1 infection, and to shed some light on the immunotherapeutic potential of γδ T cells for the treatment of HIV/AIDS.

### 5.1. Impact of HIV on γδ T Cells

In healthy individuals, Vγ9Vδ2 T cells contribute to 90–95% of the total γδ population in the peripheral blood, and the remaining 5–10% are Vδ2^neg^, such as Vδ1 and Vδ3s [72]. In HIV-infected individuals, Vγ9Vδ2 T cells are drastically depleted, and the Vδ2:Vδ1 ratio is inverted in peripheral blood [72]. Interestingly, Vγ9Vδ2 cell depletion occurs very early during the infection, and correlates with viral load and CD4+ T cell depletion [73]. HIV preferentially depletes phosphoantigen responsive Vγ9Vδ2 cells with Vγ9-Jγ1.2 TCR rearrangement [74]. Although the precise mechanism of depletion of Vδ2 cells in HIV-infected individuals is not understood, some studies indicate that α4β7 and CCR5 receptors form a complex on Vγ9Vδ2 cells, which facilitates the binding of the bV3 loop of HIV gp120 to CCR5 in the absence of expression of the CD4 co-receptor. This interaction leads to p38 kinase activation and induces apoptosis in these cells [75]. Additionally, HIV infection leads to loss of Th17 CD4+ T cells, important for maintaining epithelial barrier integrity in the gut [76]. Depletion results in dysregulation of mucosal immunity and allows microbial translocation into the circulation, resulting in systemic immune activation [77] and expansion of Vδ1 cell numbers in viremic patients [78]. Moreover, the Vγ9Vδ2 subset from HIV-infected individuals fails to proliferate or produce cytokines in response to mycobacterial infection in vitro, suggesting that Vγ9Vδ2 cells are functionally inactive [79]. This functional anergy in residual Vγ9Vδ2 cells is also characterized by their decrease in responsiveness to phosphoantigens and lytic activity toward the Daudi lymphoma cell line [80].

Vδ1 cells are enriched in the gut mucosa, where they help in maintaining tissue homeostasis. HIV infection further increases the frequency of Vδ1 cells in the mucosa, similar to the increased levels observed in peripheral blood of HIV-infected individuals [81]. Despite immunologic control of viral replication, elite controllers (EC) also display elevated levels of Vδ1 cells in the gut mucosa [82]. Few studies demonstrated that HIV-mediated disruption of gut epithelial barrier leads to translocation of gut microbiota, which activate Vδ1 T cells to produce pro-inflammatory cytokines and exacerbate the chronic inflammation [78,83]. The increase in Vδ1 T cell numbers seen in EC strongly correlated with the gut viral load, implying that viral replication in the mucosa and disruption in the gut epithelial barrier integrity and microbial translocation may contribute to enhanced Vδ1 T cell proliferation, also along with other immune activation events and disease progression. However, further studies are needed to better understand the causation and mechanism of Vδ1 T cell expansion in the gut during HIV infection and the net impact of this on disease progression.

### 5.2. Impact of γδ T Cell Perturbations on Immune Control of HIV

As previously alluded to, much of the impact of γδ T cells on immune function in health and disease is mediated through their bi-directional cross-talk with other immune cells [42]. In healthy individuals, activated γδ T cells induce the maturation and differentiation of DCs and B cells into functional APCs, but in HIV-infected individuals, this immunomodulatory capacity of γδ T cells is compromised [84]. Typically, upon activation, Vγ9Vδ2 T cells can drive the upregulation of CD80, CD86, HLA-DR, and CD40 surface expression on APCs, enhancing their capacity to induce primary αβ T cell responses [56]. HIV infection alters the ability of APCs to process and present antigens, inhibiting the ability of Vγ9Vδ2 T cells to effectively interact with and positively impact the phenotype and function of APCs [84].

HIV infection may also impair the APC function of γδ T cells and their ability to induce αβ T cell responses [85]. Activated γδ T cells produce large quantities of chemokines, including the macrophage inflammatory protein (MIP)-1α/CCL3, MIP-1β/CCL4, and CCL5/RANTES, which binds to, and can compete with the HIV coreceptor CCR5 to block HIV infection of the target cells. Again, alteration of γδ T cells in HIV infection may lead to reduced protection against new infection of target cells. Vδ2 T cells cultured in the presence of HMBPP and IL-21 express B cell-attracting chemokine CXCL13, and the CXCL13 receptor CXCR5. This promotes B cell somatic mutation, productive rearrangement, and maturation in the germinal center, thereby promoting antibody production by B cells [86,87,88]. Due to the loss of γδ T cells in HIV infected individuals, it is likely that these crucial helper functions will also be compromised.

Numerous studies have demonstrated the protective role of γδ T cells in inducing cytotoxicity in HIV-infected cells and controlling HIV replication [17,50,89]. However, γδ T cells can also contribute to negative outcomes in HIV infection. γδ T cells from HIV-infected individuals express high levels of the inhibitory receptor TIGIT, and they are the primary inflammatory driver in ART-suppressed HIV infection, and they contribute to age-associated morbidity and mortality [90]. This negative outcome is particularly highlighted in respiratory conditions, where alveolar immune cell homeostasis is disrupted in HIV-infected adults, characterized by the increased infiltration of γδ T cells and other immune cells in broncho-alveolar lavage fluid, resulting in an enhanced susceptibility of these individuals to lower respiratory tract infections [91].

### 5.3. γδ T Cell Contribution to HIV Reservoirs

Even though current ART effectively suppresses HIV replication, the integrated viral genome remains transcriptionally silent in host chromatin, representing a major challenge towards efforts to eradicate infection [92]. Resting memory CD4+ T lymphocytes have long been considered the major reservoir site for HIV [93]. However, recent studies demonstrate that other cell types can also contribute to the latent HIV reservoir, including γδ T cells [82]. 

In one study, replication-competent HIV could be obtained from highly purified Vδ2 T cells from HIV-infected people during ART, thus identifying a previously unrecognized latent HIV reservoir within Vδ2 T cells at an unexpectedly high frequency [94]. Although the precise mechanism is not known, γδ T cells can be infected by the CXCR4-tropic laboratory clone, HIV_LAI_ [80]. Possible mechanisms of infection include the binding of HIV envelope glycoproteins to the highly expressed CCR5/α4β7 receptor on Vγ9Vδ2 T cells, leading to infection through a CD4-independent pathway [75]. Alternatively, HIV infection has been shown to induce immune activation and subsequent upregulation of CD4 receptors on some γδ T cells, potentially making them more susceptible to HIV infection [94]. Preliminary findings from our group, utilizing a humanized mouse model, suggest that activated Vγ9Vδ2 cells can indeed serve as early targets for HIV infection and play a critical role in the early stages of viral dissemination [95]. Depletion of these cells during early infection would likely negatively impact immune defenses, particularly due to the important roles they serve by producing chemokines that can act as competitive inhibitors to block HIV entry and to recruit other immune effector cells to promote HIV clearance [18]. However, the increased presence and survival of activated γδ T cells, which can also serve as targets for HIV infection, could contribute to enhancement in viral production and rebound. This raises more questions about the role of γδ T cells in the initial sequelae of HIV infection and their potential contribution to the HIV reservoir.

### 5.4. Impact of Anti-Retroviral Therapy on γδ T Cells in HIV-Infection

Although ART is very effective in restoring CD4+ T cell counts and suppressing the virus below detectable levels, it fails to fully restore standard frequencies of Vγ9Vδ2 T cells in HIV-infected individuals [72]. Long-term ART partially restored the Jγ1.2 repertoire of the Vδ2 subset [74]. These Vγ9Vδ2 cells are highly activated [96] but are functionally compromised, with diminished cytokine production, cytotoxicity, and proliferation [97]. At the same time, expanded Vδ1 levels remain stable during ART in peripheral blood and mucosal sites, and they produce pro-inflammatory cytokines and express the exhaustion molecule CD279 (PD-1) [98]. Whether long-term ART impacts the number of Vδ1 T cells and/or their activation status is not clearly understood. Studies suggest that the loss of Vγ9Vδ2 cells in viral controllers is significantly lower than untreated or ART individuals, and that γδ17 cells are highly preserved. This preserved γδ population may be responsible for preventing microbial translocation and controlling chronic systemic immune activation [99,100].

### 5.5. γδ T Cells in HIV Immunotherapy

γδ T cells are the first line of defense against many pathogens, but their number and functions are severely altered in many infectious diseases, including HIV. Despite the long-term ART, γδ T cells are not reconstituted to the original frequency in HIV-infected individuals. However, in elite controllers, the Vγ9Vδ2 T cell numbers are maintained at normal levels, implying that immunotherapy using Vγ9Vδ2 T cells might recapitulate the immune status seen with those capable of naturally controlling HIV infection [99]. Several efforts were made to develop suitable methods for activating and expanding γδ T cells in vitro and in vivo. In vitro methods involve stimulating PBMCs with bisphosphonates, such as IPP, HMBPP, and zoledronate [101]. Zoledronate blocks the metabolic conversion of IPP, allowing this phosphoantigen to accumulate until stimulatory levels are reached, resulting in the selective activation and expansion of Vγ9Vδ2 T cells [101]. Bisphosphonate-mediated expansion of γδ T cells is a rapid means to generate large quantities of these cells for adoptive cell therapy. On the other hand, the delta one T (DOT) cell subtype of γδ T cells can be expanded using OKT-3 monoclonal antibody and a cytokine cocktail consisting of rIL-4, rIFN-γ, rIL-21, and rIL-1β [102]. A high yield of DOT cells could be obtained by treating PBMCs with Con-A and rIL-2 and rIL-4 [103]. Alternative strategies for expanding γδ T cells that do not respond to pAgs or N-BP involve the use of agonistic monoclonal antibodies (mAb). Using γδ TCR-specific antibodies, low levels of expansion of Vδ1 and Vδ2 T cells has been achieved, but it has not been very successful to date. However, 20.1, an agonistic Ab specific for CD277 (a member of BTN3 subfamily), mimics pAg-induced Vγ9Vδ2 cell activation. This antibody may simulate a conformational change in the CD277 molecule to activate and expand Vγ9Vδ2 T cells [104].

In vivo approaches include zoledronate and rIL-2 combination treatment (Table 2) to induce Vγ9Vδ2 T cell expansion and maturation [105]. Expanded Vγ9Vδ2 T cells belong mainly to the central memory and effector memory subgroups. Increased DC maturation and HIV-specific CD8+ T cell responses were observed in zoledronate-treated patients [105]. Very limited adverse events of zoledronate have been found, highlighting the safety of the zoledronate treatment. Moreover, the expansion of Vγ9Vδ2 T cells in HIV-infected patients could also improve tumor immunity and enable better control of opportunistic pathogens. However, information concerning the impact of zoledronate treatment on viral RNA and CD4+ T cell levels is lacking and should therefore be addressed in future clinical trials [105].

Specifically, in the case of HIV-infected individuals, phosphoantigen-responsive Vγ9Vδ2 T cells can be recovered by supplementation with IL-18, which promotes inflammasome formation and the robust activation and expansion of Vγ9Vδ2 cells in vitro [106]. Differences in the capacity to recover and expand γδ T cells from those who initiate ART during early vs. late stages of infection might be an obstacle for their use in immunotherapy. On the other hand, γδ T cells from non-HIV infected individuals can be grown efficiently, and therefore have been considered for their use in allogeneic immunotherapy settings. Importantly, transplantation of allogeneic γδ-T cells can result in successful engraft without inducing graft-versus-host disease (GVHD), since their mechanism of antigen recognition is MHC-independent [107]. Adoptive transfer of ex-vivo-expanded γδ T cells in HIV-infected individuals is an attractive HIV therapeutic strategy to consider.

Importantly, the potential for inducing immune reconstruction syndrome (IRS) [112], a phenomenon associated with immune reconstitution and production of proinflammatory cytokines by activated T cells should be recognized when considering γδ T cells in HIV therapy. Regarding toxicity, the combined therapeutic use of bisphosphonate and IL-2 has been used with modest success in cancer patients to promote the in vivo expansion of γδ T cells, with relatively minor adverse effects, including fever, injection site soreness, nausea, and diarrhea, being reported [113]. However, some cancer studies reported decreased γδ T cell responses during extended bisphosphonate/IL-2 therapy regimens due to the development of anergy [113]. Repeated administration of bisphosphonate/IL-2 may be worth considering as a therapy for chronic HIV, but the potential for driving functional anergy in γδ T cells would need to be carefully evaluated. Since treating γδ T cells with rapamycin (targeting both MTORC1 and MTORC2) has been shown to reduce the anergy, the inclusion of this factor as part of combination therapy may be a reasonable alternative to overcome some of the potential problems in γδ T cell-based immunotherapy [114].

## 6. Into the Unknown with γδ T Cells

While γδ T cells play a crucial role in controlling cancer, infection, and mediating tissue homeostasis in response to injury, the underlying mechanisms are not fully understood. The specific processes used by γδ T cell subsets to recognize “danger” are unique when compared to conventional αβ T cells. Even with decades of ART, the frequency and function of the γδ T cell population do not become fully restored [115]. However, in vivo administration of zoledronate to HIV-infected individuals has been shown to improve γδ T cell numbers and function [105]. Importantly, a complete understanding of the therapeutic potential of this approach for controlling HIV infection has yet to be fully studied.

Increasing interest in γδ T cell-based immunotherapy for cancer is driving innovative and novel approaches to harness their cytotoxic potential for an HIV cure. The recent clinical success of chimeric antigen receptor (CAR) T cells in treating leukemia and lymphoma, and the use of tumor-infiltrating lymphocytes in solid tumor patients gives new hope for using similar strategies to genetically modify γδ T cells to address their dysfunction during HIV infection, or to express HIV infection-specific CAR, targeting highly conserved HIV epitopes to capitalize on their natural cytotoxic capacity. Although in vitro studies have demonstrated the HIV latency clearing capacity of Vδ2 cells [110], further studies are warranted to replicate these findings in vivo and to determine the exact mechanisms of their target recognition. Animal studies using such approaches as humanized mouse models or non-human primates may prove critical to addressing questions related to the in vivo potency of γδ T cells in targeting tissue-resident HIV reservoirs. Nevertheless, the demonstrated protective function of γδ T cells in the numerous preclinical studies shown to date have been encouraging. As more researchers focus their attention to better understand these specialized immune cells, increases in the “knowns” of the γδ T cells will surely enhance the possibilities of their “unknowns”.

## Figures and Tables

**Table 1:** Distribution and function of γδ subtypes.

γδ Subtypes	Vδ1	Vδ2	Vδ3
Distribution	Dermis, spleen, liver, gut epithelia, lung, peripheral blood, and other mucosal sites	Peripheral blood and lymphatics	Peripheral blood, liver, and gut
Function	Maintain epithelial tissue integrity by recognizing stress-induced MICA/B [4] Major source of IL-17 [14] Lyse autologous tumor [15] Opsonization of CMV through CD16 induce IFN-γ response [16]. Cytotoxicity against HIV infected cells through NKG2C triggering [17]. Recognize lipid antigens presented by CD1d [4].	Recognize phosphoantigens produced by various microbes and transformed host cells in an MHC-independent manner and induce cytotoxicity [1]. Promote Th1 response by IFN-γ and TNF-α production [13]. Produce chemokines CCL-4 and CCL-5, which block HIV co-receptor CCR-5 [18]. Modulate B cell and DC maturation [19]. Enhance NK cell cytotoxicity via CD137 Interaction [20]. Antibody-dependent cellular Cytotoxicity [18].	Function through cognate interaction with HLA-A2 and CD1d [7]. Expand in peripheral blood of renal and stem cell transplant recipients with CMV reactivation and B cell chronic lymphocytic leukemia [9]. Modulate B cell and DC maturation [10,11].

**Table 2:** HIV immunotherapy studies using Vδ2 T cells.

Treatment	Participant Status	Result	Reference
**Adoptive transfer of zoledronate + IL-2 expanded PBMCs**	HIV infected humanized mice	No impact of Vδ2 T cell on HIV induced CD4+ depletion or plasma viremia	[108]
**HMBPP + IL-2 injected in Macaque**	SHIV infected Macaque	Expansion and activation of Vδ2 T cell. Increase in Env specific antibody but no impact on viral load.	[109]
**Pamidronate + IL-2 expanded PBMCs**	Human HIV + ART	Inhibition of HIV replication in vitro. PAM expanded Vδ2 T cells suppress p24 production by infected CD4+ T cells in the presence of vorinostat.	[110]
**Ex vivo Vδ2 T cells**	Human HIV + ART	CD16 activation on Vδ2 T cells from HIV infected individuals on ART promote ADCC in vitro.	[111]
**Ex vivo IPP + IL-18**	Human HIV + ART	IL-18 improves IPP induced Vδ2 activation, proliferation, and degranulation in HIV infected individuals	[106]
